# Advances in the application of colorectal cancer organoids in precision medicine

**DOI:** 10.3389/fonc.2024.1506606

**Published:** 2024-12-03

**Authors:** Yanan Zhang, Ruoyu Meng, Dan Sha, Huiquan Gao, Shengxi Wang, Jun Zhou, Xiaoshan Wang, Fuxia Li, Xinyu Li, Wei Song

**Affiliations:** ^1^ The First Clinical Medical College of Shandong University of Traditional Chinese Medicine, Jinan, Shandong, China; ^2^ Department of Oncology, Zibo Hospital of Traditional Chinese Medicine, Zibo, China; ^3^ Department of Minimally Invasive Comprehensive Treatment of Cancer, Shandong Provincial Hospital Affiliated to Shandong First Medical University, Jinan, Shandong, China; ^4^ Department of Radiotherapy, The Affiliated Yantai Yuhuangding Hospital of Qingdao University, Yantai, China

**Keywords:** colorectal, organoid, precision medicine, drug screening, single-cell sequencing, gene editing, CRC

## Abstract

Colorectal cancer (CRC) ranks among the most prevalent gastrointestinal tumors globally and poses a significant threat to human health. In recent years, tumor organoids have emerged as ideal models for clinical disease research owing to their ability to closely mimic the original tumor tissue and maintain a stable phenotypic structure. Organoid technology has found widespread application in basic tumor research, precision therapy, and new drug development, establishing itself as a reliable preclinical model in CRC research. This has significantly advanced individualized and precise tumor therapies. Additionally, the integration of single-cell technology has enhanced the precision of organoid studies, offering deeper insights into tumor heterogeneity and treatment response, thereby contributing to the development of personalized treatment approaches. This review outlines the evolution of colorectal cancer organoid technology and highlights its strengths in modeling colorectal malignancies. This review also summarizes the progress made in precision tumor medicine and addresses the challenges in organoid research, particularly when organoid research is combined with single-cell technology. Furthermore, this review explores the future potential of organoid technology in the standardization of culture techniques, high-throughput screening applications, and single-cell multi-omics integration, offering novel directions for future colorectal cancer research.

## 1 Introduction

### 1.1 Background

#### 1.1.1 Incidence of colorectal cancer and its global implications

Colorectal cancer (CRC) is the third most common malignancy and the second leading cause of cancer-related deaths worldwide, with approximately 1.926 million new cases and 904,000 deaths reported in 2022 (1). Despite advances in treatment, 30-50% of early-stage patients still experience recurrence (2). The global incidence of CRC continues to rise, with projections suggesting 3.2 million new cases and 1.6 million deaths by 2040. Developed countries, such as the United States and Western European nations, face particularly high incidence rates, likely due to dietary habits, lifestyle, and aging populations. Current treatments include surgery, radiotherapy, and chemotherapy, supplemented by emerging immunotherapy and targeted therapies (1). However, challenges in targeting efficiency, drug resistance, and treatment efficacy persist, partly due to the lack of models that accurately replicate the *in vivo* disease environment. Organoid technology, which closely mimics *in vivo* tumor characteristics, offers significant potential for both research and clinical applications (3–5).

#### 1.1.2 Concept and importance of precision medicine

Precision medicine is a healthcare model that customizes medical treatment based on an individual’s genetic, environmental, and lifestyle factors. The core of precision medicine lies in using individualized information to prevent, diagnose, and treat diseases, aiming to provide personalized healthcare. This concept emphasizes the application of advanced biotechnologies, particularly genomics, proteomics, metabolomics, and other multi-omics, to achieve more accurate disease diagnosis and treatment (6, 7). Especially in CRC, precision medicine predicts patient responses to specific treatments by identifying particularly genetic mutations, such as *KRAS*, *BRAF*, and *PIK3CA*, while also reducing unnecessary treatment side effects (8).

Precision medicine not only focuses on effective disease treatment but also prioritizes prevention and health management, with the goal of improve treatment outcomes, reduce side effects, and lower healthcare costs (1). Unlike traditional healthcare models that rely on generalized population data, precision medicine seeks to deliver “customized” solutions for each patient (9).

The significance of precision medicine can be summarized in five key areas: 1) Improvement of treatment efficacy: by analyzing individual patients’ genomes and biomarkers, more accurate drug dosages and combinations can be determined, enhancing treatment effectiveness. For example, exome sequencing can identify specific tumor mutations, guiding the selection of optimal targeted therapies (10). 2) Reduction of side effects: by predicting patient responses to medications, physicians can minimize adverse reactions by avoiding ineffective or harmful drugs. 3) Disease prevention: precision medicine facilitates early disease detection and prevention through genetic screenings, enabling the identification or prediction of specific disease risks and allowing for lifestyle adjustments to mitigate morbidity risk (4). 4) Optimization of healthcare resource allocation: precision medicine reduces trial-and-error approaches, expedites diagnostic and treatment decisions, and conserves healthcare resources, thereby lowering costs. This is particularly crucial for optimizing resource allocation (11). 5) Promotion of medical research: precision medicine drives research into disease mechanisms, aids in discovering new therapeutic targets, and supports the development of personalized diagnostic and treatment strategies (12).

### 1.2 Overview of organoid technology

#### 1.2.1 Definition and application of organoids

Organoids are typically derived from adult stem cells, embryonic stem cells, or induced pluripotent stem cells and are cultured *in vitro* under specific signaling and environmental conditions. These structures replicate the multicellular composition and physiological functions of their original organs in a laboratory setting, offering a more physiologically relevant model than traditional two-dimensional cell cultures.

**Figure 1:**
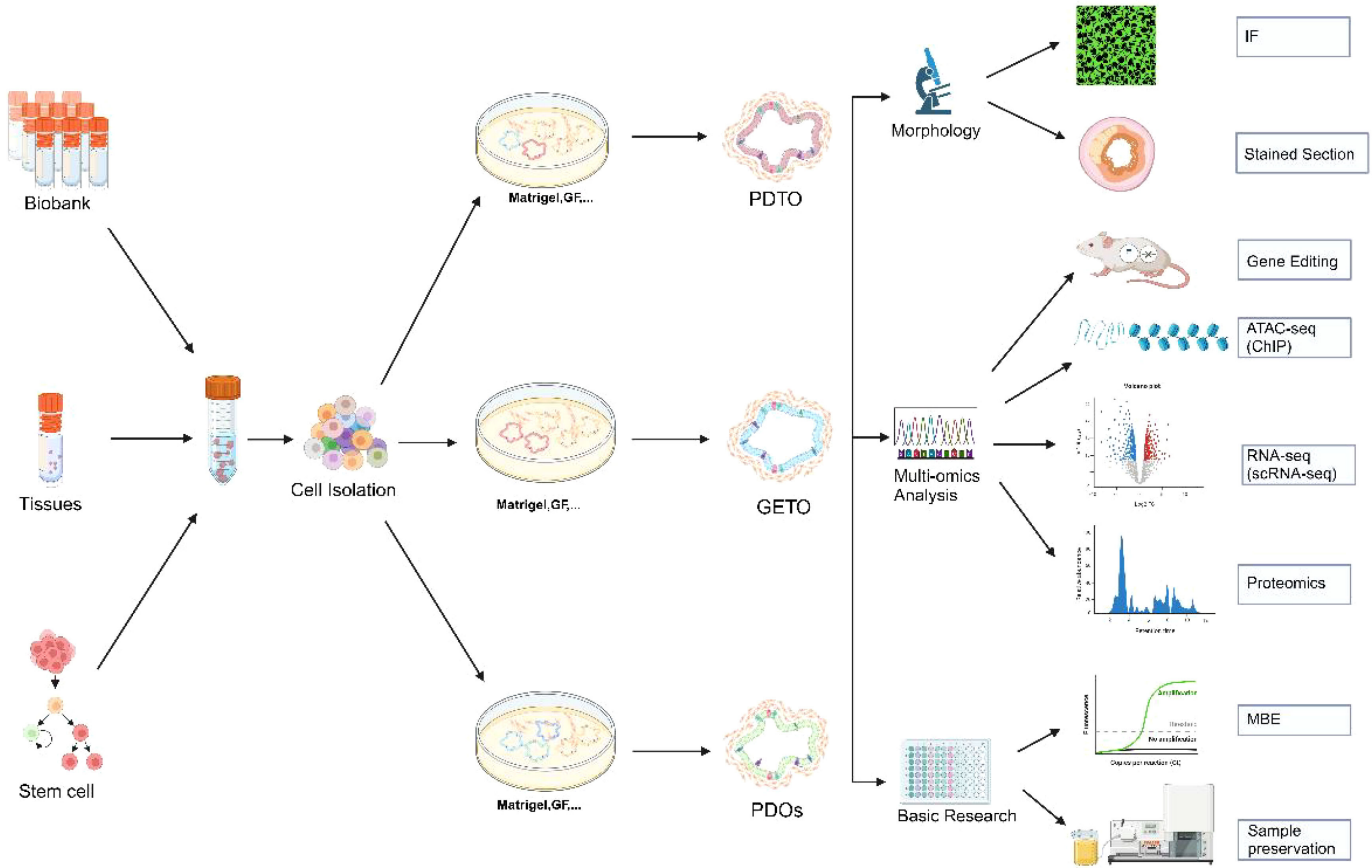
Establishment of colorectal cancer organoids and its application in morphology, multi-omics analysis and basic research. Biobank, human tissue-derived, and stem cell-derived cells can be cultured into colorectal organoids. These organoids are applicable for morphological observation, multi-omics analysis (including gene editing, ATAC-seq analysis, single-cell transcriptome analysis, and proteomic analysis), and basic research. PDTX, Patient-derived tumor xenograft model; GETO, Genetically modified tumor organoids; PDTO, Patient-derived organoids. Created with Biorender.com (accessed on 15 August 2024).

Organoid technology has vast potential in cancer research, genetic disease modeling, and the exploration of infectious pathogenesis (13). In cancer research, organoids can recreate the tumor microenvironment, serving as an effective tool for targeted drug screening and personalized therapy. In genetic disease research, patient-derived organoids can mimic the effects of gene mutations, advancing the study and treatment of rare diseases. Patient-derived organoids (PDOs) are invaluable for predicting patient treatment responses *in vitro* and have been successfully applied in various cancers, including colorectal (14), gastric (15), lung (8), and liver cancers (16). The development of organoid technology, particularly the construction of PDOs from tumor tissues, has significantly enhanced its value in precision medicine. By recreating the tumor microenvironment, organoid technology preserves the structural and functional characteristics of donor tissues, making it a crucial platform for studying tumor biology and developing personalized therapies (**Figure 1**).

#### 1.2.2 Potential and advantages in colorectal cancer research

Intestinal tumor organoids can differentiate into almost all intestinal cell types, including stem cells, Paneth cells, goblet cells, endocrine cells, and epithelial cells (17). Currently, there are two primary methods for constructing patient-derived tumor organoids (PDTOs): differentiation of induced pluripotent stem cells (iPSCs) or direct derivation from tumor tissues. However, the success rate of iPSC-derived PDOs varies significantly depending on the tumor type, and the complexity of the procedure can reduce efficiency. Additionally, PDOs generated from iPSCs often lack the tumor microenvironment’s complexity, being predominantly epithelial, whereas iPSC-derived organoids may include both epithelial and mesenchymal cells (18). Therefore, the future trend in organoid research lies in the culture techniques of PDTOs directly derived from tumor tissues, supplemented with cytokines and extracellular matrices. Directly deriving organoid cultures from tumor tissues is relatively straightforward, as both surgical specimens and biopsy samples can be used for colorectal cancer organoid cultures.

The culture process involves isolating cancer stem cells from colorectal cancer(CRC) tissues, embedding these cells in stromal gels, and adding specific media for expansion and passage, followed by experimental studies (19)(**Figure 1**). Although organoids are not fully developed organs, embedding intestinal crypts or tumor cells in an extracellular matrix (ECM) containing growth factors and inhibitors successfully replicates the *in vivo* stem cell niche at the base of intestinal crypts, enabling long-term proliferation and differentiation without genetic modifications. Consequently, organoids have become an ideal model system for studying intestinal homeostasis and CRC, preserving the molecular characteristics of donor tissues in terms of structure and function (10). Organoids exhibit a high degree of congruence with their source tissues in various biological characteristics, including gene composition, protein expression, biomechanical properties, and biochemical properties (17, 20). Early and late passage organoids maintain essentially identical gene mutations, demonstrating their genetic stability in long-term culture (10). By constructing organoids with *APC*, *KRAS*, and *TP53* mutations, researchers can simulate the progression of CRC and evaluate the effectiveness of various treatment strategies (21). Organoids also offer advantages such as short cultivation time, ease of operation, high culture efficiency, transmissibility, freezing capability, and ease of genetic modification, rendering them ideal models for achieving precision tumor therapy.

Numerous research has confirmed that colorectal cancer organoids, as a novel tool for in-depth tumor research, drug screening, and therapeutic sensitivity prediction, hold immense potential in tumor precision medicine. This review summarizes the development of colorectal cancer organoids and recent advances in precision medicine, while also discussing current challenges.

## 2 Construction and culture of colorectal organoids

### 2.1 Sources of organoids

#### 2.1.1 Human tissue sources

The sources of colorectal cancer organoids primarily include two categories: human tissue and stem cells. Direct cell isolation from human tissues is a common method for constructing colorectal cancer organoids. This approach typically involves extracting cells from a patient’s tumor tissue and forming organoid models through *in vitro* three-dimensional culture (**Figure 1**). These organoids retain the genomic features and morphological characteristics of the original tumor, positioning them an essential tool for studying tumor heterogeneity and treatment response (20, 22).

Tumor heterogeneity research: Organoids derived from human tissues can reflect the heterogeneity of different cell populations within a tumor, aiding researchers in understanding the differential responses of various cell types to treatment (20). For instance, different tumor cell subtypes may exhibit varying sensitivities to the same drugs, which is crucial for developing personalized treatment strategies (5).

Drug screening and resistance studies: Using patient-derived organoids allows for the testing of multiple drugs under laboratory conditions and the identification of resistance mechanisms (3, 5). This not only accelerates new drug development but also provides a method for optimizing drug combinations tailored to specific patients (4, 12).

#### 2.1.2 Stem cell sources

Stem cells, particularly induced pluripotent stem cells (iPSCs) and adult stem cells, offer another significant pathway for constructing colorectal cancer organoids. Especially with intestinal stem cells, organoids can be formed under appropriate culture conditions (23). By adding specific growth factors, such as Wnt, Noggin, and R-spondin, to the culture medium, stem cell-derived organoids can maintain long-term proliferation and develop structures resembling the intestine (24, 25). These stem cells can be induced by specific culture conditions and signaling molecules to differentiate into organoids with tumor characteristics *in vitro* (26).

Application of iPSCs: iPSCs can be reprogrammed into any cell type, and specific tumor mutations can be introduced through gene editing, generating organoids with distinct genomic features. This is vital for examining the role of genetic mutations in tumorigenesis (23).

Utilization of adult stem cells: Adult stem cells isolated from normal or cancerous tissues can also be used to generate organoids. These organoids can be maintained *in vitro* for extended periods, exhibiting growth and differentiation properties resembling those of *in vivo* tissues, thereby positioning them an ideal model for studying tumor development and treatment (11, 27, 28).

### 2.2 Organoid culture techniques

#### 2.2.1 3D culture technology

Culture technology for colorectal cancer organoids is a core aspect of organoid research, providing a more physiologically relevant model by mimicking the *in vivo* environment for three-dimensional cell growth and differentiation. Three-dimensional (3D) culture technology forms the foundation for organoid construction, utilizing biological scaffolds or matrix gels that place cells in a three-dimensional environment to support tissue characterization (9, 29).

Matrigel culture: The most used 3D culture technique is Matrigel or similar matrix gel-based cultures. This method allows cells to self-assemble into organoid structures by providing a support analogous to the extracellular matrix (ECM). The application of Matrigel mimics the natural microenvironment of cells *in vivo*, enhancing cell-matrix interactions to better replicate *in vivo* conditions (9).

Bioscaffolding technology: In addition to matrix gels, bioscaffolding materials are frequently used in 3D culture. These scaffold materials, typically made of natural or synthetic polymers, can be tailored in terms of porosity, rigidity, and other properties to accommodate the growth requirements of various cell types. This approach has broad applications in tissue engineering and regenerative medicine (30).

Self-assembling organoids: Cells can self-assemble into organoids under scaffold-free or low-scaffold conditions by precisely controlling cell density and culture conditions. This self-assembly process relies on cell-to-cell signaling and interactions, generating tissues that more closely resemble physiological states (31). **Table 1** provides a detailed comparison of the various models used in cancer research, demonstrating the strengths and limitations of each.

**Table 1:** Comparison of tumor cell lines, spheroid culture, PDX, GETO, and PDTO models based on key metrics.

Feature	Tumor Cell Lines	Spheroid Culture	PDX	GETO	PDTO
Expense	+	+	++	++	++
Time consuming	+	+	+++	++	++
Culture success rate	+++	+++	+	+++	+++
Genome editing	+++	+++	–	+++	+++
Organization structure	–	+	+++	++	++
Tumor biological property	+	++	+++	+++	+++
Tumor heterogeneity	–	+	+++	++	++
Tumor microenvironment	–	+	+++	++	++
Gene stability	+	+	++	++	++
High throughput screening of drugs	+++	+++	–	+++	+++
Personalized treatment	–	–	++	+++	+++
Establishment success rate	+++	–	+	+++	++
Maintenance	+++	++	+	+++	+++
Growth speed	+++	+++	+++	++	+
Expansion	+	–	++	++	++
Reproducibility	+++	–	+	+++	+++
Representativeness	+++	–	+	++	+
Tumor immune microenvironment	+	–	+++	+	+++
Complexity	–	+	–	+	+
Large scale application	+	–	–	++	+
Normal controls	–	–	+	++	+
Genetic tumor model	–	–	–	+	+
Immune cell infiltration	–	–	+++	+++	+++
Patient-Derived variant representation	–	+	+++	+++	+++
Model scalability	+++	+	+	++	++
Ethical considerations	++	++	–	++	+++

PDTX, Patient-derived tumor xenograft model; GETO, Genetically modified tumor organoids; PDTO, Patient-derived tumor organoid model. “+” indicates a low score, “++” a medium score, “+++” a high score, and “-” unsuitable.

#### 2.2.2 Microenvironmental simulation

The success of organoid technology relies heavily on the effective simulation of the *in vivo* microenvironment, which includes the combined modulation of physical, chemical, and biological factors to sustain organoid growth, differentiation, and functionality (32).

Physical environment simulation: In organoid cultures, physical parameters such as oxygen concentration, shear stress, and mechanical pressure are precisely modulated to mimic the real environment cells encounter *in vivo*. As an illustration, hypoxic conditions are often used to replicate the hypoxic microenvironment of tumor tissues to study tumor growth and metabolic properties (33).

Control of the chemical environment: The regulation of media composition and growth factors is crucial for organoid growth. By adding or removing specific growth factors and chemical signals, the differentiation and maturation of organoids can be directed. This method has been widely used to investigate cell signaling pathways and drug responses (34, 35).

Reproduction of biological interactions: Microenvironmental simulations also involve the interactions of immune cells, stromal cells, and microbial communities with organoids. By co-culturing with other cell types or introducing microbial communities around organoids, researchers can more comprehensively explore cellular interactions and their impact on disease processes (6, 36, 37).

### 2.3 Characterization and validation of organoids

#### 2.3.1 Morphological analysis

Characterization and validation of organoids are essential to ensuring they accurately mimic physiological and pathological states *in vivo*. Morphological analysis is one of the fundamental methods for characterizing organoids, providing detailed observations of their structural and organizational features through microscopy. The formation of typical crypt-villus structures in organoids can be observed, allowing for the assessment of growth and differentiation under various conditions (38).

Microscopic observation: Light and electron microscopy are commonly used to assess the overall morphology and cellular substructures of organoids. Light microscopy enables observation of the three-dimensional structure and growth of organoids, while transmission electron microscopy provides higher resolution details of cellular structures (39).

Tissue sections and staining: Organoid sections, combined with HE staining and immunohistochemistry, can reveal internal tissue structures and cell distribution.

*In vivo* imaging technology: *In vivo* imaging, through fluorescence labeling and confocal microscopy, allows real-time observation of organoid growth and development without destruction, making it particularly suitable for studying cell migration and differentiation dynamics.

#### 2.3.2 Multi-omics analysis including genome and transcriptome

Multi-omics analyses offer a comprehensive view of the molecular characteristics of organoids, covering genomic, transcriptomic, proteomic, and metabolomic dimensions.

Genomic analysis: Whole genome sequencing (WGS) and targeted gene sequencing can confirm whether the genetic characteristics of organoids align with the original tissues, validating the genetic accuracy of organoids, especially in cancer research and genetic disease modeling (5). Whole-genome sequencing of organoids derived from 30 patients revealed that these organoids retained key mutations in *APC*, *KRAS*, and *TP53*, and exhibited abnormal activation of the Wnt and PI3K signaling pathways, further validating the effectiveness of the organoid model (40–42).

Transcriptome analysis: RNA sequencing (RNA-seq) reveals the full spectrum of gene expression in organoids, enabling in the identification of key genes and signaling pathways associated with disease states or therapeutic responses (43, 44). Transcriptomic data comparison enables assessment of the similarity between organoids and *in vivo* tissues in terms of gene expression levels.

Proteomic and metabolomic analyses: Techniques like mass spectrometry identify and quantify proteins and metabolites in organoids, providing essential insights into the functional state and metabolic activity of cells. These data are vital for evaluating the functional and metabolic integrity of organoids. The application of single-cell technology in organoid research will be discussed later (45, 46).

### 2.4 The role of biospecimen banking in organoid research

#### 2.4.1 Establishment and maintenance of biospecimen banks

Biospecimen repositories are vital in organoid research, offering essential resources and technical support for tumor biology research and personalized medicine. By systematically collecting and managing biological samples from various patients, sample banks not only enhance the scope of organoid research but also contribute to the advancement of precision medicine. Establishing a biospecimen repository requires careful consideration of sample quality and availability, encompassing key aspects such as collection, storage, management, and quality control (5, 22) (**Figure 1**).

Sample collection and processing: Tumor tissues obtained through clinical surgery or biopsy are processed quickly to prevent degradation. Samples are typically stored frozen in liquid nitrogen or at -80°C.

Storage and management systems: Modern biobanks utilize advanced database systems to manage and track sample information, recording each sample’s origin, pathological characteristics, genomic data, and clinical information to ensure accurate and prompt access to data.

Quality control and ethical considerations: Regular quality checks, such as DNA/RNA integrity and viability testing, are essential for maintaining sample quality. Additionally, obtaining informed consent from patients is necessary for the use of samples to ensure the ethical legitimacy of the research.

#### 2.4.2 Sample banks in organoid studies

Sample repositories provide various applications for organoid research, facilitating the exploration of disease mechanisms and the development of therapeutic strategies (**Figure 1**).

Disease modeling: Patient tumor tissues from sample banks can be used to construct multiple organ models that accurately reflect the biological characteristics of a patient’s tumor, serving as crucial tools for studying the mechanisms of various cancer types.

Drug screening and development: Organoid models in sample banks enable high-throughput drug screening to evaluate the efficacy and safety of new drugs and identify potential resistance mechanisms (22).

**Figure 2:**
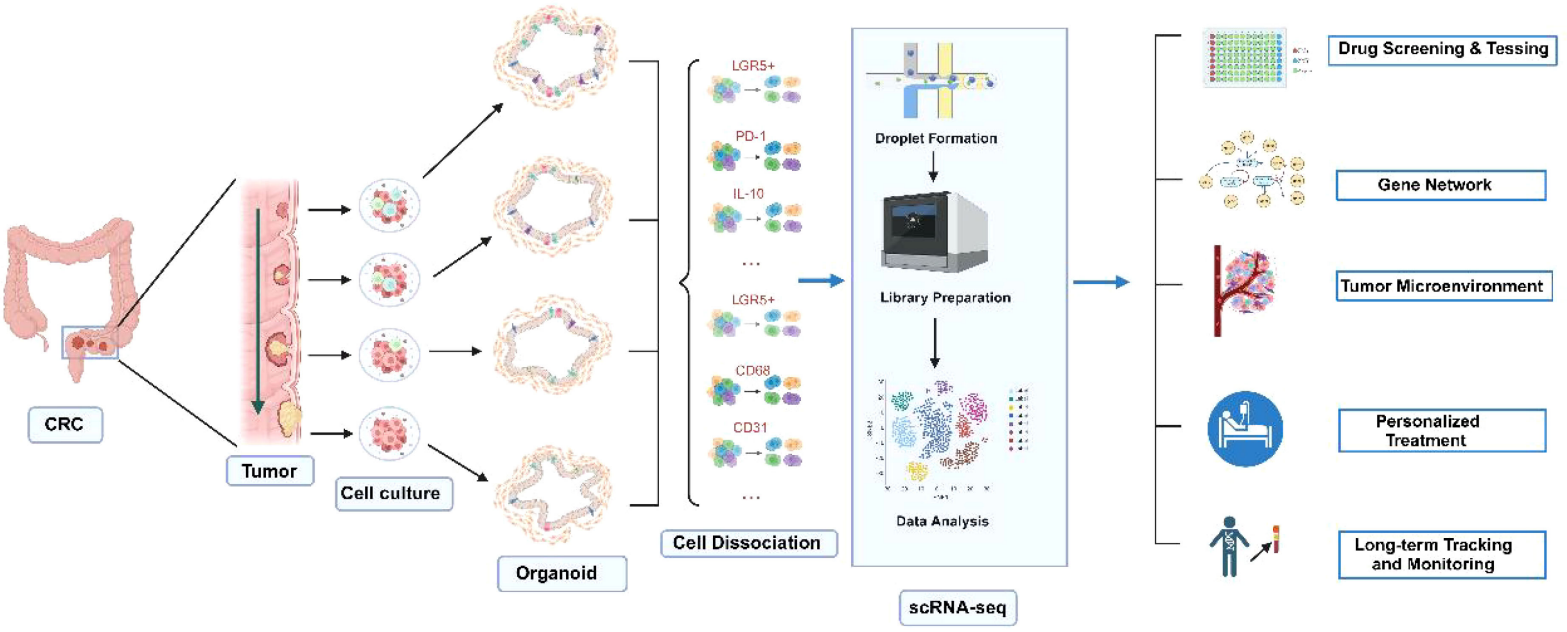
Applications of CRC organoids combined with single-cell sequencing technology. Normal and tumor tissues are used to generate corresponding organoids. By applying single-cell sequencing technology, the roles of various cell types in CRC development can be elucidated. This approach is valuable for drug screening and testing, gene function studies through gene editing, personalized treatment, as well as long-term tracking and monitoring. Created with Biorender.com (accessed on 15 August 2024).

Multi-omics analysis: Organoid models can be subjected to multi-omics analyses, including genome, transcriptome, and proteome, contributing to the identification of disease biomarkers and therapeutic targets, thereby providing valuable data for precision medicine (**Figure 2**).

#### 2.4.3 Integration of sample banking and organoid studies

The integration of sample banking and organoid research is a key driver of personalized and precision medicine.

Personalized medicine: The diversity of patient samples provided by sample banks makes personalized medicine feasible. By simulating a patient’s pathological characteristics through organoid models, researchers can validate the effectiveness and safety of individualized treatment plans, advancing the realization of personalized treatment.

Collaborative innovation and resource sharing: Sample banks facilitate collaboration and resource sharing among research institutions, enabling scientists to access a broader range of sample types and data, fostering interdisciplinary research and innovation. This synergistic model can also improve the efficiency of research translation.

Clinical translation and application: Organoid research supported by sample banks accelerates the translation of basic research into clinical applications. The application of organoid modeling in clinical trials enhances the success rate of new drug development and reduces R&D costs.

A comparison of various metrics for the construction of tumor cell lines, spheroid culture, PDX, GETO, and PDTO models is presented in **Table 1**.

## 3 Application of organoids in colorectal cancer research

### 3.1 Disease modeling

#### 3.1.1 Simulation of pathological features of colorectal cancer

Organoids not only retain the heterogeneity of patient tumors by mimicking key pathological features of colorectal cancer, such as multiple cell types, varying differentiation states, and complex tumor microenvironments, but also exhibit biological behaviors and drug responses *in vitro* that closely resemble those observed *in vivo*. By decellularizing human colonic tissue and reintroducing organoid cells carrying *APC*, *KRAS*, and *TP53* mutations, researchers successfully recapitulated the development of colorectal cancer (42). Further transcriptomic analysis revealed significant activation of the Wnt/β-catenin and PI3K/AKT signaling pathways in the organoids, which closely matched the gene expression profiles observed in actual patient samples (21, 47, 48).

Cellular heterogeneity: Colorectal cancer organoids effectively reproduce the cellular heterogeneity of tumors, which is crucial for understanding tumor progression and treatment response (49). These organoid models facilitate in-depth studies of the interactions among different cell types during cancer progression and provide essential data for developing personalized treatment strategies (50, 51).

Tumor microenvironment: Organoid technology also enables the replication of the tumor microenvironment’s complexity, including key factors such as angiogenesis and immune cell infiltration. This capability allows researchers to gain a better understanding of how microenvironmental factors influence tumor growth and treatment response, leading to the development of more effective therapeutic strategies (52).

#### 3.1.2 Application of genome editing in disease modeling

**Figure 3:**
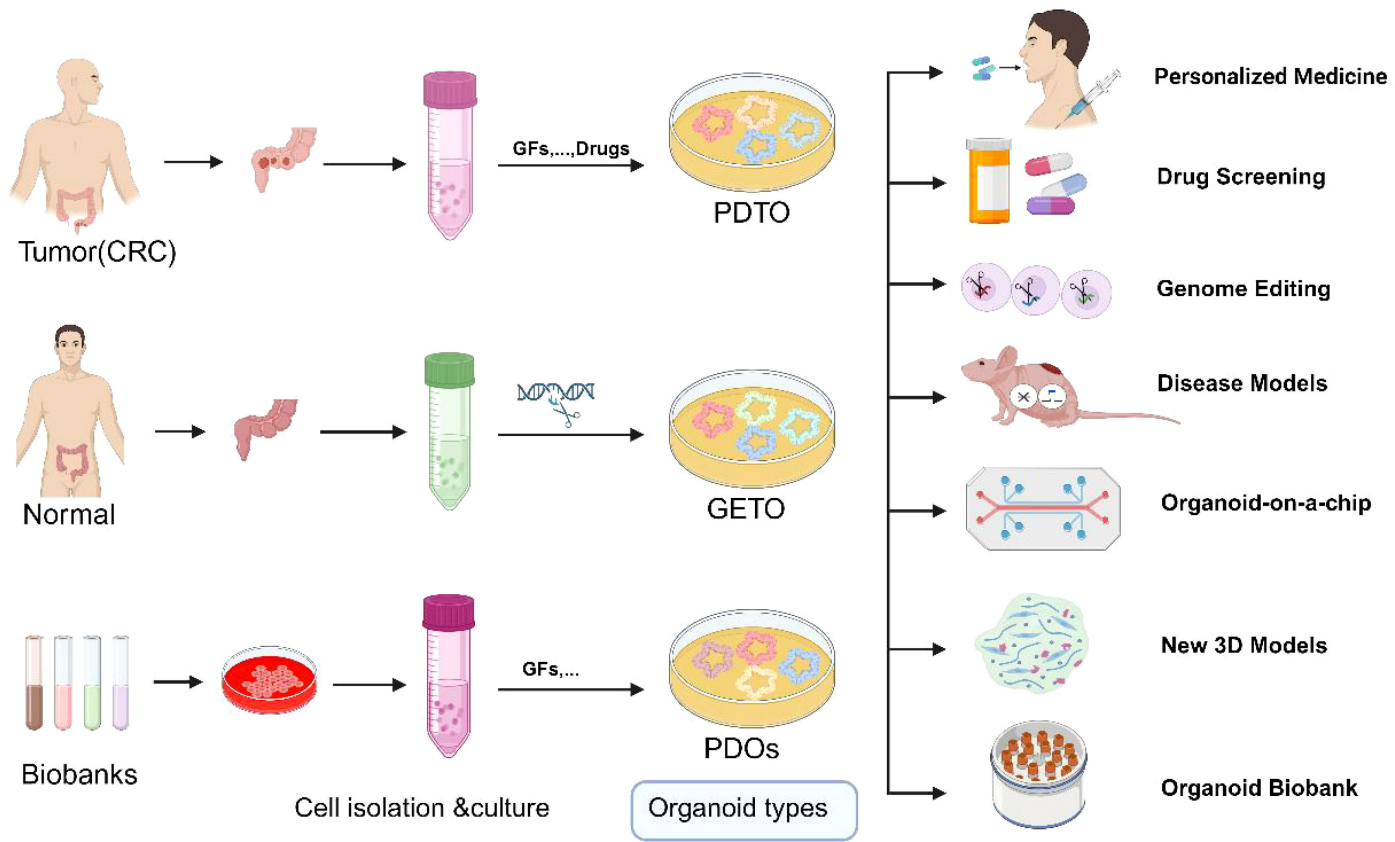
Schematic representation of the application of normal, tumor organoids and Biobank. GETO and PDTO organoid models play a pivotal role in advancing personalized medicine, drug screening, gene editing, disease modeling, organoid-on-a-chip platforms, the development of novel 3D models, and the establishment of organoid biobanks. PDTX, Patient-derived tumor xenograft model; GETO, Genetically modified tumor organoids; PDTO, Patient-derived organoids. Created with Biorender.com (accessed on 15 August 2024).

Creation of mutation models: Using CRISPR-Cas9 technology, researchers can precisely introduce specific gene mutations (knock-in) into organoids to study their roles in colorectal cancer initiation and progression (38). This approach is pivotal for deepening our understanding of cancer’s genetic underpinnings and for developing novel targeted therapies (**Figure 3**).

Functional genomics research: Systematic knockdown or activation of specific genes using CRISPR technology allows the exploration of gene functions in cancer. This approach not only aids in identifying potential therapeutic targets and biomarkers but also provides molecular data to support precision medicine. Using CRISPR-Cas9 technology, human intestinal organoids were sequentially edited to introduce *APC*, *TP53*, *KRAS*, and *SMAD4* mutations, successfully modeling the multistep progression of colorectal cancer (52). More than 50 gene-edited organoids were transplanted into mice, resulting in the formation of invasive adenocarcinomas (12). Genomic sequencing indicated that these organoids exhibited typical chromosomal instability (CIN) and highlighted the synergistic action of key genes during tumor progression (38) (**Figure 2**).

Research on drug resistance mechanisms: Genome editing can also be utilized to investigate drug resistance mechanisms. By simulating various drug exposure environments in organoids, researchers can elucidate how genetic changes lead to resistance to chemotherapy and targeted therapies, thus providing new directions and strategies to overcome drug resistance (11, 53) (**Figure 2**).

Gene editing in organoids using adenoviral and lentiviral transduction, combined with Cre-lox and CRISPR-Cas9 technologies, enables the investigation of specific genetic events in cancer development. Additionally, the integration of CRISPR technology with single-cell RNA sequencing techniques, such as Perturb-seq and CROP-seq, allows the elucidation of gene functions at the single-cell level and the study of multicellular interactions within the tumor microenvironment during tumorigenesis and development (54).

### 3.2 Drug screening

#### 3.2.1 High-throughput drug screening

Organoids have emerged as an indispensable tool for drug screening due to their genetic and epigenetic stability and their highly similar gene expression profiles compared to primary tissues. Tumor organoids effectively capture tumor heterogeneity, meeting the demand for large-scale drug screening within short timeframes (55, 56) (**Figure 3**).

The Clevers Laboratory conducted the first medium-scale drug screening using its colorectal cancer (CRC) organoid biobank, demonstrating the feasibility of using patient-derived organoids (PDOs) for such tests and highlighting their potential as clinically relevant biomarkers of therapeutic response (57). Van De Wetering pioneered preclinical high-throughput drug screening with organoid libraries, successfully establishing PDOs from both primary and metastatic colorectal cancer lesions (22). They performed extensive comparisons of the genomic profiles of these organoids with their original tumors, showing that the organoids closely resembled the genetic change profiles of the tumors and enabling the identification of gene-drug correlations. For instance, CRC organoids with *RNF43* mutations were sensitive to Porcupine inhibitors, while those with *Kras* mutations exhibited resistance to tyrosine kinase receptor inhibitors (22).

High-throughput drug screening was performed on 22 tumor organoids derived from 20 colorectal cancer patients, testing over 200 compounds. The results revealed that organoids harboring the KRASG13D mutation exhibited significant sensitivity to MEK inhibitors, while their response to standard chemotherapy drugs, such as 5-FU and irinotecan, was highly dependent on specific genetic backgrounds (41).

Kondo et al. screened 2,427 drugs using CRC patient-derived organoids, discovering that the organoids displayed varying sensitivities to different drugs (58). Additionally, Du et al. developed a higher-throughput organoid drug screening platform capable of efficiently completing screening tests for thousands of drugs, further validating the enormous potential of organoids in developing new therapeutic regimens (59). Shen et al. identified the Kruppel-like factor 5 inhibitor ML264 by conducting *in vitro* drug screening with patient-derived CRC organoids. ML264 overcame drug resistance by restoring apoptosis in CRC organoids and re-sensitizing them to oxaliplatin (60). USUI et al. constructed organoids expressing high levels of LGR5 and CD44 to study tumor cell drug resistance mechanisms and screen colorectal cancer drugs. Their findings indicated that combining Hedgehog signaling inhibitors (AY9944 and GANT61) with anticancer drugs could inhibit tumor cell stemness, reduce organoid colony formation, and reverse drug resistance, significantly improving patient outcomes (61).

The OncoTrack Collaboration established a large biobank comprising 106 tumors, 35 organoids, and 59 xenografts, testing 16 clinical drugs on both *ex vivo* and *in vivo* models (62). The study by Shen et al. reaffirms the potential of organoid models for high-throughput drug screening and contributes to advancing precision therapies.

#### 3.2.2 New drug development and personalized treatment

Human tumor cell lines remain the primary targets for anticancer drug research. However, one of the significant obstacles in new drug development is translating laboratory results into clinical trials, largely because traditional 2D tumor cell lines do not accurately reflect the physiological characteristics of primary tumors. Organoids facilitate this process and reduce the costs associated with new drug development. Tumor tissues and normal tissues from the same patient can be cultured into organoids, enabling simultaneous drug sensitivity and toxicity tests. This dual approach allows for the identification of drugs that specifically kill tumor cells without being toxic to normal cells, thereby reducing adverse drug reactions (**Figure 3**). For instance, in drug development, tumor organoids can be used to test a drug’s target-specific killing ability, while organoids derived from liver, heart, and kidney tissues can assess hepatotoxicity, cardiotoxicity, and nephrotoxicity, respectively. This strategy ensures the selection of drugs that target tumor cells without harming normal tissues. Furthermore, by simulating *APC*, *KRAS*, *TP53*, and *PIK3CA* mutations in organoids, researchers have successfully screened and validated several targeted therapies specific to different genotypes. Genomic and transcriptomic analyses revealed that these drugs significantly inhibited the activation of MYC and Wnt signaling pathways, thereby suppressing organoid growth. This paper demonstrates that integrating patient-specific organoids with genomic data can effectively optimize personalized treatment strategies and expedite the development of new drugs (41). Researchers have developed a powerful drug screening system that identified 34 drugs with anti-CRC properties among 335 drugs. Drugs were classified into five modes of action through transcriptome analysis: differentiation induction, growth inhibition, metabolic inhibition, immune promotion response, and inhibition of cell cycle (3).

### 3.3 Research on drug resistance mechanisms

#### 3.3.1 Mechanisms of chemotherapy resistance

Drug resistance remains one of the major challenges in colorectal cancer treatment, highlighting the importance of understanding the underlying mechanisms to develop more effective therapeutic strategies. Chemotherapy is a standard treatment for colorectal cancer, but many patients develop resistance during therapy, resulting in diminished efficacy.

Expression of multidrug resistance pumps: Cancer cells often excrete chemotherapeutic drugs by upregulating multidrug resistance proteins such as P-glycoprotein (P-gp) and multidrug resistance-associated proteins (MRP), thereby reducing intracellular drug concentrations (63). Organoid models can accurately mimic changes in the expression of these proteins, providing valuable insights into their role in drug resistance (53). The study found that organoids harboring the KRASG12D mutation were resistant to standard chemotherapy regimens, whereas those with the KRASG13D mutation exhibited varying degrees of drug sensitivity (64).

Apoptotic escape: Chemotherapeutic drugs typically induce cancer cell death by triggering apoptosis, but some cancer cells evade this process by regulating apoptotic signaling pathways, such as by upregulating Bcl-2 family proteins (65). Organoids retain the apoptotic regulatory properties of tumors, thereby serving as an ideal platform for the investigation of this escape mechanism (66).

Enhanced DNA repair: Cancer cells can resist chemotherapy-induced DNA damage by enhancing their DNA repair capabilities, often through upregulating key DNA repair enzymes like PARP (67). Organoid models are valuable for elucidating the role of these repair pathways in drug resistance and for devising novel strategies to overcome resistance (68).

#### 3.3.2 Analysis of resistance to targeted therapy

Targeted therapies inhibit tumor growth by interfering with specific molecular pathways, but drug resistance remains a significant challenge.

Genetic mutations and altered therapeutic targets: Targeted drugs often focus on specific genetic mutations, but cancer cells can acquire new mutations or alter existing targets, leading to resistance. As an example, *EGFR* and *KRAS* mutations render targeted therapies ineffective in some colorectal cancer patients (69). The combined inhibition of PD-1, BRAF, and MEK has been used to treat colorectal cancer patients and organoid models with the BRAFV600E mutation (63, 70). Although the initial treatment achieved significant antitumor effects, some patients and organoids developed resistance during the later stages of therapy. Molecular analysis indicated that upregulation of EGFR signaling was the primary cause of this resistance (70).

Activation of paracrine signaling pathways: When primary signaling pathways in cancer cells are inhibited, these cells may continue to grow by activating alternative pathways. When the EGFR pathway is blocked, cancer cells may maintain growth by activating the MET or HER2 pathways (71). Organoid technology can be employed to study the redundancy of these signaling pathways and their role in drug resistance (52, 72).

Impact of the tumor microenvironment: The tumor microenvironment, including interactions between immune cells and stromal cells, significantly affects the efficacy of targeted drugs. Organoids can mimic these complex microenvironmental interactions, enabling researchers to investigate how drug resistance might be overcome by modulating the microenvironment (32).

### 3.4 Organoid chip technology

#### 3.4.1 Principles of organoid chips

Organochips are miniature cell culture systems derived from the application of microfluidic devices in cell culture and bioanalysis. By utilizing micro-nanofluidic processing technology, a multifactorial and complex reaction system is integrated onto a chip, where the entire process of culture, experimentation, and analysis is completed under precise control. The objective is to replicate essential cellular structures and functions of human organs on a chip. Organoids on a chip serve as a platform to investigate the processes and mechanisms driving tumor progression and treatment resistance, including tumor cell migration and invasion, tumor heterogeneity, and interactions with the tumor microenvironment (TME), encompassing stromal components, immune cells, endothelial cells, and chemokines (27).

Microarrays are widely used for 3D high-throughput organoid culture. Microwell structures facilitate the rapid aggregation of stem cells, a crucial step in generating uniformly sized organoids. These structures not only organize the culture system into regular arrays, enhancing throughput, but also provide geometric constraints and microenvironmental cues that guide organoid development and maturation. Recent studies have aimed to optimize microwell substrate materials to further improve culture efficiency.

#### 3.4.2 Applications in colorectal cancer research

Organoid microarray models have been developed to explore the interaction between tumor cells and blood vessels in colorectal cancer. Research on the activin-ALK 7 pathway revealed its key role in insufficient vascularization of tumors, affecting drug delivery and chemotherapy efficacy (73). Hu et al. combined organoid technology with microfluidics to develop a chip with integrated superhydrophobic microwell arrays, achieving drug sensitivity predictions in just one week (74). Maxim et al (75). developed a targeted organoid sequencing technology (TORNADO-seq) for monitoring the expression of large genes to assess cellular phenotypes in organoids. TORNADO-seq enables the analysis of cell mixtures and their differentiation status within the intestinal system, providing valuable insights into drug mechanisms of action. A gut-on-a-chip integrated with transepithelial electrical resistance (TEER) sensors and electrochemical sensors was successfully used to simulate the human intestinal microenvironment and monitor cellular physiology (76).

#### 3.4.3 Advantages of organoid chips

A significant advancement in organoid microarray technology is the “fusion” of normal tissue organoids with tumor organoids to form “organ microchips.” This technology allows not only the observation of cell-cell interactions within the tumor but also the examination of overall organ-to-organ correlations. Such a system simulates the process of tumor metastasis *in vivo* and enables the visual detection of drug pharmacokinetics and pharmacodynamics, thereby enhancing drug screening success rates and mitigating ethical concerns associated with animal models. A GOC integrated with a carcinoembryonic antigen (CEA) biosensor was developed, it can detect CEA through antigen antibody specific binding, with high selectivity, excellent stability, and good reproducibility (77).Furthermore, the development of high-throughput automated organoid microarrays will contribute to the standardization of organoid culture processes.

## 4 Organoid applications in precision medicine

Organoids offer substantial potential in precision medicine, especially in the formulation of individualized treatment strategies. By accurately replicating the 3D architecture and microenvironment of a patient’s tumor, organoid technology provides a robust *in vitro* model for personalized therapy. Notable applications include:

Individualized drug screening: leveraging high-throughput screening of patient-derived organoids to identify the most effective therapeutic regimens.

Predicting treatment response: utilizing organoid-based assays to evaluate various drug combinations, enabling the prediction of patient responses and the optimization of treatment plan.

Disease modeling and gene editing: applying gene editing techniques and disease modeling in organoids to investigate the functional roles of specific genes in disease pathogenesis.

### 4.1 Development of individualized treatment strategies

#### 4.1.1 Application of patient-specific organoids

Immunotherapy research is progressing rapidly, and the integration of organoid technology offers a powerful tool to advance our understanding of tumor immunity and escape mechanisms. This approach has the potential to significantly accelerate the development of immunotherapies and enhance the efficacy of precision cancer treatments. It is well established that bidirectional communication between tumor cells and both cellular components (e.g., fibroblasts, endothelial cells, immune cells) and non-cellular components (e.g., extracellular matrix) within the tumor microenvironment (TME) plays a crucial role in tumor initiation and progression (**Figures 1**, **2**).

However, conventional organoid cultures typically contain only tumor cells and lack stromal and immune cells, necessitating the exogenous addition of immune cells to study tumor-immune cell interactions. Co-culture of organoids and immune cells reconstructs the tumor immune microenvironment, overcoming this limitation (36, 78). As an illustration, researchers constructed an organoid biobank from colorectal cancer patient tumor tissues, with these organoids retaining the genomic characteristics of the original tumors, including mutations in *APC*, *TP53*, and *KRAS*. High-throughput screening revealed that these patient-specific organoids could accurately predict responses to standard chemotherapy and targeted therapies. For instance, organoids carrying PIK3CA mutations showed significant sensitivity to PI3K inhibitors, providing strong support for the development of individualized treatment plans (22).

Organoid models simulating the tumor microenvironment (TME) can be categorized into initial TME models and reconstructed TME models, depending on the inclusion of exogenous immune and stromal cells. Initial TME models encompass microfluidic culture and air-liquid interface (ALI) culture. Microfluidic culture can preserve endogenous immune cell subpopulations from tumor tissues while allowing the addition of exogenous immune cells, such as CAR-T cells. In contrast, ALI culture is more practical due to its simplicity and the absence of specialized equipment requirements. They found that these cells could specifically kill EGFRvIII+ colorectal cancer organoids without affecting normal tissues, suggesting that the organoid-immune cell co-culture system could assess CAR-mediated tumor-specific cytotoxicity. Additionally, studies have demonstrated that peripheral T cells can acquire intraepithelial lymphocyte characteristics—including morphology, markers, and motility—when co-cultured with intestinal organoids (79, 80). However, further research is needed to fully align the immune microenvironment of organoids with *in vivo* conditions (81).

#### 4.1.2 Predicting treatment response and optimizing treatment regimens

Tumor heterogeneity is a hallmark of cancer biology and a significant contributor to treatment failure. As a highly heterogeneous solid tumor, colorectal cancer presents a challenge in that only a small fraction of patients responds favorably to treatment (32). Colorectal cancer organoids offer a more accurate reflection of patient treatment sensitivity, providing reliable experimental data and new insights for clinical precision therapy. While organoids are an ideal platform for antitumor drug testing and personalized treatment, a standardized protocol for drug sensitivity testing has yet to be established. The most common approach involves assessing organoid cell viability at therapeutic doses (**Figure 2**).

In 2015, Weeber et al. (82) demonstrated that organoids from metastatic colorectal cancer patients retain 90% of somatic mutations with a DNA copy number correlation coefficient of 0.89, confirming their genetic fidelity to the original tumor. Organoids accurately predicted treatment responses in colorectal and gastroesophageal cancers with a positive predictive value of 88% and a negative predictive value of 100% (83), and they also effectively forecast radiotherapy sensitivity, enabling personalized treatment (84).

Organoids have shown high accuracy in predicting chemotherapy outcomes (85). Tiriac et al. (81) linked chemotherapy responses in pancreatic tumor-derived organoids with patient outcomes, while Ganesh et al. (86) found a significant correlation between organoid sensitivity and progression-free survival. Rectal cancer organoids also predicted chemotherapy and radiotherapy responses, with *TP53* and *BRAF* mutations linked to higher radiotherapy resistance, confirmed clinically (86). However, Ooft et al. (87) faced significant challenges in predicting the efficacy of various chemotherapy regimens. The observed discrepancies between organoid sensitivity and clinical outcomes may be attributed to the low success rate of organoid culture and the suboptimal quality of the samples.

In summary, colorectal cancer (CRC) organoids have demonstrated a robust ability to predict treatment efficacy, underscoring their significant potential for clinical application. By tailoring individualized treatment regimens based on organoid-derived predictions, it is anticipated that drug resistance can be reduced, overtreatment avoided, and adverse effects minimized. This makes organoid technology a promising tool for integration into prospective clinical trials on a broader scale (**Figure 2**).

### 4.2 Application of single-cell technology in precision medicine

#### 4.2.1 Cellular-level interpretation of individualized clinics

Single-cell technology reveals heterogeneity and dynamic changes among cells by analyzing genomic, transcriptomic, and epigenetic features at the individual cell level, providing detailed cellular information for disease diagnosis and treatment strategies in precision medicine.

Identify cell types associated with individual therapeutic response: Single-cell technology can identify cell subpopulations closely associated with therapeutic response. By analyzing single-cell RNA sequencing data from tumor samples, key cell types and gene expression patterns influencing drug efficacy can be identified (88) (**Figure 3**). The presence of immunosuppressive cell subpopulations in tumors has been found to predict the effectiveness of immunotherapy. Tumor-reactive T cells were generated by co-culturing organoids with patient-derived peripheral blood lymphocytes (6). These T cells demonstrated the ability to specifically target and kill colorectal cancer organoids with defective mismatch repair (dMMR). Further single-cell RNA sequencing analysis uncovered the diversity of T-cell subsets and their correlation with tumor-killing efficacy (6) (**Figure 2**).

Customized treatment plan optimization: Leveraging single-cell data, clinicians can refine and optimize personalized treatment plans. By gaining detailed insights into the responses of various cell types within a patient’s tumor, treatment regimens can be precisely tailored to the tumor’s unique characteristics, thereby enhancing treatment efficacy and minimizing adverse side effects (89).

#### 4.2.2 Early detection of tumor progression and recurrence

Single-cell technology has important applications in early tumor detection. By recognizing specific cancer biomarkers, single-cell technology can diagnose the disease in its early stages, making it particularly useful for detecting tiny lesions and metastases that are difficult to identify using traditional methods. For instance, researchers utilized multivariate single-cell mass spectrometry flow analysis of heterogeneous organoids to uncover the specific signaling networks of different cell types involved in tumor progression. The analysis revealed that cells within the tumor microenvironment possessing certain post-translational modifications (PTMs) exhibited increased invasiveness and a higher potential for recurrence (90). The abnormal expression of microRNA-21 (miR-21) is closely related to the pathogenesis of cancer, but in the early stage of cancer, its serum concentration is very low. To solve this problem, a nano-genosensor based on the free-standing MWCNT electrode was successfully developed for measuring miR-21 (91).

Monitoring the risk of tumor recurrence after treatment: Single-cell analysis can monitor changes in the tumor microenvironment post-treatment and identify early signs of recurrence. By tracking dynamic changes in residual tumor cells, treatment strategies can be adjusted in a timely manner to prevent disease recurrence (92).

#### 4.2.3 Assisted gene therapy and immunotherapy

Single-cell technology enables precise evaluation of gene editing effects, providing in-depth insights into editing efficiency and off-target effects. By analyzing single-cell genomic data from edited cells, researchers can verify the specificity and accuracy of gene editing to optimize gene therapy strategies (88).

Combining single-cell technology with organoids allows for the simulation of the tumor immune microenvironment and the study of immune cell-tumor cell interactions. This approach is crucial for developing and optimizing immunotherapy strategies, such as checkpoint inhibitors and CAR-T cell therapy. The dynamics of immune cells in organoids are closely related to clinical patient responses. A genome-wide CRISPR screen identified the Wnt-FZD5 signaling pathway as a viable drug target for RNF43 mutant pancreatic tumors. Investigators developed antibodies that specifically target FZD5 and validated their anti-tumor efficacy in both organoid and mouse models (93).

By integrating organoid and single-cell technologies, precision medicine is progressively achieving the goal of individualized diagnosis and treatment. This integration not only enhances our understanding of tumor biology but also provides patients with more precise and effective treatment options, laying the foundation for improving overall cancer treatment outcomes.

### 4.3 Challenges and opportunities for clinical translation

#### 4.3.1 Data integration and analysis

Organoid research generates vast amounts of multi-omics data, including genomic, transcriptomic, proteomic, and metabolomic information. The cellular heterogeneity of colorectal cancer organoids was comprehensively analyzed using single-cell RNA sequencing. The study revealed significant differences in gene expression and drug response across various clones within the organoids (40). Integrating these heterogeneous data types is a complex task requiring standardization and integration to achieve a comprehensive understanding of biological systems (94).

Data Standardization: Given the diversity and heterogeneity of organoid data, it is crucial to develop uniform data standards and formats. This includes standardizing the entire process of data acquisition, storage, and analysis (53).

Data integration platforms: Developing flexible data integration platforms and tools is essential for effectively combining multiple data types and helping researchers identify new biological associations and potential therapeutic targets. Data analysis is a critical step in extracting meaningful insights from large datasets, and advanced analytical tools will accelerate the clinical application of organoid technologies.

Establishing a good data integration platform first requires accurate provision of patient clinical information, and secondly, clear and detailed records of organoid culture, passage, and cryopreservation should be kept for data extraction and further analysis. In addition, accurately recording and analyzing the morphology and structure of organoids is also an important aspect, as they can reflect their growth and development status. For example, the area of colorectal cancer organoids can indicate their survival ability. However, currently there are few automated tools and instruments used to analyze the growth status of organoids, and they still mainly rely on visual analysis or immunofluorescence staining observation after sectioning. With the rapid development of artificial intelligence, a series of excellent algorithms have emerged that can obtain detailed information and decipher image content. Li analyzed organ chips based on deep learning for data analysis, automation, and image digitization (95). More and more researchers are seeking methods to quantify and analyze organoids through AI, in order to achieve three-dimensional imaging, intelligent recognition, and analysis of organoids.

Artificial intelligence and machine learning: The application of Artificial Intelligence (AI) and Machine Learning technologies allows for a degree of automation in processing and analyzing organoid data, helping to identify complex biological patterns and predict drug responses.

Big data analytics: Utilizing big data analytics methods enables researchers to extract patterns and trends from large-scale datasets, which is particularly important for understanding disease mechanisms and developing personalized treatment plans.

#### 4.3.2 Application in clinical trials

Organoid technology offers a physiologically relevant model for drug development and testing, significantly enhancing the efficiency and success of clinical trials. A study evaluated the efficacy of a combination therapy involving PD-1, BRAF, and MEK inhibitors in BRAFV600E mutant colorectal cancer. The clinical efficacy and safety of this therapy were validated by successfully translating findings from organoid studies into clinical trials (70). A clinical trail from Denmark enrolled 90 patients with metastatic colorectal cancer following progression on or after standard therapy and treated with a panel of drugs from organoids models based-drug screening. The results revealed that the primary endpoint was met, as half of the patients were without progression at two months (96). This clinical trial strongly proved that cancer patients may benefit from functional testing using tumor-derived organoids.

Drug screening and toxicity testing: Organoids can be used for high-throughput drug screening and toxicity testing, aiding in the identification of potential drug candidates and the optimization of drug combinations (53).

Individualized drug testing: With patient-derived organoids, clinical trials can more accurately mimic an individual patient’s tumor response, providing data to support personalized therapy. In a phase 2, single-center, open-label, non-comparative study (ClinicalTrials.gov, register NCT03251612), 90 patients with metastatic tumors who progressed after treatment were subjected to organoid culture, of which 44 were successfully established and drugs with anticancer activity were screened *in vitro*, and at least one drug was used for treatment. The results showed that 17 patients (50%) achieved a progression free survival of two months, exceeding the expected level (14 of 45;31%). This strongly suggests that *in vitro* sensitivity testing of organoids can effectively guide the selection of clinical anti-tumor drugs and improve patient benefits (97).

Biomarker identification: Organoids can be used to identify and validate biomarkers that predict therapeutic response, thereby optimizing patient selection strategies and improving the success of clinical trials (4, 98).

Simulation in early trials: By simulating the efficacy and toxicity of a drug in an organoid model, potential issues can be identified, reducing the risk of failure in the early stages of clinical trials (99).

The use of organoid technology allows for faster and more efficient clinical trials while significantly improving the success rate of new drug development. These advantages make organoids an indispensable tool in precision medicine research. However, despite their potential, challenges remain in translating organoid applications to clinical settings.

## 5 Limitations and future directions of organoid technologies

### 5.1 Technical limitations

#### 5.1.1 Complexity and reproducibility of models

Complexity: The complex structure of organoids makes their culture and maintenance challenging. Variations in experimental conditions can lead to significant differences in organoid growth and differentiation, affecting the reliability of experimental results (12, 32).

Reproducibility: Inter-laboratory reproducibility is a critical issue due to the diversity of organoid sources and culture conditions. Different laboratories may use varying media, scaffold materials, and growth factors, resulting in greater variability in outcomes (96).

#### 5.1.2 Differences from the internal environment

Simulation of the microenvironment: Organoids cannot fully replicate the complex microenvironment present *in vivo*, including interactions with the vascular, nervous, and immune systems. This limitation hinders the application of organoids in studying multicellular interactions and systemic diseases (32).

Physiological dynamic changes: Organoids are typically static models that lack the dynamic physiological changes observed *in vivo*, such as blood flow, mechanical stress, and metabolic activity. This deficiency limits their ability to model certain physiological processes (6, 14). ssQuadruple mutant organoids (APC, KRAS, TP53, SMAD4) did not exhibit metastatic behavior despite forming aggressive tumors in mice. This finding suggests that cancer development and progression are not solely driven by genetic mutations but also require the interplay of additional molecular and environmental factors (100).

To summarize, the limitations of organoid technologies are fourfold: (1) the success rate of organoid construction is often compromised by bacterial contamination, tumor characteristics, and specific culture media, making it difficult to establish organoids for some types; (2) organoids established from a single time-point sample only represent the state at that moment, whereas tumors *in vivo* are dynamically evolving; (3) organoids are inadequate at fully modeling the tumor microenvironment; and (4) organoids do not replicate the collaborative interactions between multiple organs *in vivo*, limiting their ability to provide insights into systemic biological processes.

### 5.2 Future directions

Although organoid models offer new perspectives for understanding carcinogenesis and developing therapeutic strategies, they still have inherent limitations. Compared to animal models, organoids lack functional vascular, nervous, and immune systems, which restricts their use in simulating the *in vivo* environment. Cancer development and therapeutic response involve complex processes such as vascularization, local hypoxia, and immune system activity, which organoids cannot fully replicate. For example, the standardization of culture techniques is crucial for the growth of tumor tissue-derived organoids and the accuracy of experimental results (101). Challenges such as preventing “contamination” in normal tissue-derived organoids and successfully introducing vascular systems into organoids remain unresolved. Additionally, extracellular matrix gels (e.g., Matrigel) may impede drug penetration, affecting the application of organoids in drug screening. These gels also suffer from batch-to-batch variability and lack components of the tumor microenvironment (TME) (102, 103).

#### 5.2.1 Integration with other cutting-edge technologies

Microfluidics: The combination of organoids with microfluidics can provide a dynamic fluidic environment that mimics blood flow and nutrient transport *in vivo*. This integration makes organoids more physiologically relevant for drug screening and toxicity testing (104).

Artificial intelligence: The use of artificial intelligence and machine learning to analyze organoid-generated data enables the identification of complex biological patterns and drug responses, thereby accelerating the development of new drugs and personalized treatment regimens.

#### 5.2.2 Standardization and large-scale production of organoids

Standardization: Developing uniform culture standards and protocols will improve the reproducibility of organoid experiments. This includes standardizing medium composition, scaffold materials, and culture conditions, which will help reduce inter-laboratory variation. To achieve standardization in organoid culture, it is necessary to clarify the sampling requirements for each type of organ, and standardize the composition of the culture medium and culture conditions as much as possible; And after successful cultivation, the consistency between the properties of the organoid and the parent tissue was determined through methods such as IHC and NGS. Taking colon cancer organoids as an example, for surgical removal of sample tissue, it is recommended that the size of fava beans be appropriate; For microscopic examination and puncture sample tissues, it is recommended to take at least 3 tissue samples with forceps or 3 tissue samples with coarse needles. The collected tumor tissue samples must be quickly stored in sterile tubes containing specialized preservation solution for tumor samples, and quickly transported to the laboratory at around 4°C for preservation. Growth factors such as Y-27632 to the culture medium should be added in the culture medium to maintain cell viability. It is recommended to use immunohistochemistry methods to identify tumor organoids, such as HE,CDX-2,STAB-2,CK8/18,etc (78, 105).

Scale-up production: High-throughput organoid culture technology needs to be developed to achieve large-scale production, meeting the demands of drug screening and clinical research. This advancement will not only enhance efficiency but also reduce research costs.

By addressing the limitations of current technologies and exploring new development directions, organoid technologies will continue to play a pivotal role in biomedical research, driving precision medicine toward broader clinical applications.

## 6 Conclusion

The application of colorectal cancer organoid technology in precision medicine has shown great potential. Organoid models can highly mimic multiple biological features of the original tumor, including gene expression, protein profile, metabolic activities and other aspects of the characteristics, thus providing a physiologically relevant *in vitro* model for disease research and personalized treatment. Through organoid culture, researchers can gain a deeper understanding of the pathophysiological characteristics of colorectal cancer and study key issues such as tumor heterogeneity, drug screening, and drug resistance mechanisms. These models can not only simulate the three-dimensional structure of tumors, but also reproduce the complex interactions of the tumor microenvironment, thus providing a solid foundation for new drug development and treatment optimization. In recent years, the combination of organoid technology with genome editing, single-cell technology, microfluidic technology and other cutting-edge technologies has further promoted the development of organoid research and enhanced the breadth and depth of its application in precision medicine.

However, despite the remarkable progress of organoid technology in colorectal cancer research, its application still faces many challenges. Technical complexity, standardization issues, and inter-laboratory reproducibility issues are all key factors affecting the widespread application of organoid research. In addition, organoids still have some limitations in mimicking the *in vivo* environment, such as the lack of a complete vascular system, nervous system, and immune system functions, which limits their potential application in studying systemic diseases. Therefore, future research should focus on solving these technical difficulties and enhancing the standardized and scaled production capacity of organoids by further combining cutting-edge technologies such as artificial intelligence and machine learning, so as to make their role in precision medicine more comprehensive and effective.

Overall, the development of organoid technology in colorectal cancer research is promising. By overcoming the existing technological limitations, organoids are expected to become an important tool for precision medicine, providing reliable support for the development of individualized treatment plans and new drug development. This process requires not only in-depth advancement of scientific research, but also multidisciplinary synergistic innovation to accelerate the clinical translational application of organoid technology.
